# Long-term survival and health-related quality of life in patients with severe acute respiratory distress syndrome and veno-venous extracorporeal membrane oxygenation support

**DOI:** 10.1186/s13054-021-03821-0

**Published:** 2021-11-29

**Authors:** Jonathan Rilinger, Klara Krötzsch, Xavier Bemtgen, Markus Jäckel, Viviane Zotzmann, Corinna N. Lang, Klaus Kaier, Daniel Duerschmied, Alexander Supady, Christoph Bode, Dawid L. Staudacher, Tobias Wengenmayer

**Affiliations:** 1 Department of Medicine III (Interdisciplinary Medical Intensive Care), Medical Center, Faculty of Medicine, University of Freiburg, Freiburg, Germany; 2 Department of Cardiology and Angiology I, Heart Center Freiburg University, Faculty of Medicine, University of Freiburg, Hugstetterstr. 55, 79106 Freiburg, Germany; 3 Institute of Medical Biometry and Statistics, University Medical Center Freiburg, Faculty of Medicine, University of Freiburg, Freiburg, Germany; 4 Heidelberg Institute of Global Health, University of Heidelberg, Freiburg, Germany

**Keywords:** ECMO, Extracorporeal membrane oxygenation, Acute respiratory distress syndrome, Outcome, Survival, Long-term, Quality of life

## Abstract

**Background:**

There is limited information about the long-term outcome of patients suffering from acute respiratory distress syndrome (ARDS) supported with veno-venous extracorporeal membrane oxygenation (VV ECMO). Most studies focused on short- to mid-term follow-up. We aimed to investigate long-term survival and health-related quality of life (HRQL) in these patients.

**Methods:**

We report retrospective data from a single-centre registry of patients with severe ARDS treated with VV ECMO at the Interdisciplinary Medical Intensive Care Unit at the Medical Centre, University of Freiburg, Germany, between 10/2010 and 06/2019. Follow-up data of all patients that survived the index hospitalisation were collected by telephone interviews from 02/2020 till 09/2020. Long-term survival, HRQL (Short-Form Health Survey-36 (SF-36), St. Georges Respiratory Questionnaire (SGRQ), Hospital Anxiety and Depression Scale (HADS)) and the return to work rate were documented.

**Results:**

In total, 289 patients were treated with VV ECMO during the study period (median age 55 years, 67% males, hospital survival 45%). After a median duration of 3.9 years, follow-up assessment was complete in 94 of 129 hospital survivors (73%). Fifty-three patients completed the HRQL assessment. Hospital survivors showed a high 6- and 12-month survival rate (89% and 85%, respectively). Estimated survival rate of those discharged alive from ICU was 68.5% (95%-CI 56.9–80.1%) after 9.7 years. These patients reported high levels of HRQL (median SF-36 total score 73) and only few pulmonary (median SGRQ total score 19) and mental limitations (median HAD-D score 2 and HAD-A score 3). In total, 80% of the patients were able to resume employment.

**Conclusion:**

This analysis of VV ECMO patients showed favourable long-term survival and high levels of HRQL suggesting promising prospects for VV ECMO survivors.

**Graphical Abstract:**

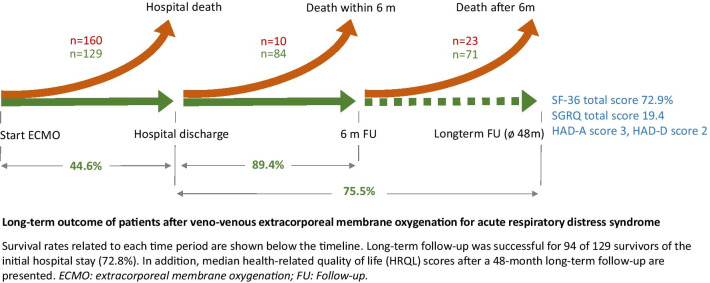

**Supplementary Information:**

The online version contains supplementary material available at 10.1186/s13054-021-03821-0.

## Background

Patients with severe acute respiratory distress syndrome (ARDS) may benefit from veno-venous extracorporeal membrane oxygenation (VV ECMO) support [1–3]. A substantial increase in the use of ECMO support has been recorded over the recent years [4]. Nevertheless, the mortality of these patients remains very high [2]. In addition, patients suffer from complications as a result of their underlying disease or as a direct consequence of ECMO support, such as secondary infections, bleedings, thromboses and embolisms [5, 6]. Moreover, patients surviving complex intensive care treatment including severe ARDS therapy and ECMO support are often severely compromised even after discharge and at risk to subsequently die in the further course [7].

It is difficult to predict long-term survival of individual patients. Future quality of life is often of major interest for the patients, relatives and ICU teams. The resource-intensive and extended course of these patients, often prone to serious complications, may lead to a high level of emotionality within the treatment teams with a potential impact on therapy decisions. More evidence about long-term survival and long-term quality of life would therefore be of great value for appropriate therapy management. However, most VV ECMO outcome studies only focus on hospital survival or a short- to mid-term outcome after 6 or 12 months, respectively [7–11].

We performed an analysis of long-term survival, long-term health-related quality of life (HRQL) and the rate of return to work with an extended follow-up period in ARDS patients supported with VV ECMO. Furthermore, we analysed factors associated with hospital and mid-term survival.

## Methods

### Study population

We report retrospective data from a single-centre registry of adult patients with severe ARDS according to the Berlin definition (Horowitz index < 100 mmHg) [12] supported with VV ECMO. VV ECMO was initiated in cases of severe hypoxic respiratory failure or hypercapnia despite invasive mechanical ventilation as suggested by ELSO guidelines [13].

All patients treated at the Interdisciplinary Medical Intensive Care Unit at the Medical Centre, University of Freiburg, Germany, from October 2010 through June 2019 were registered. Follow-up data of all patients surviving the index hospitalisation were collected by standardized telephone interviews from February 2020 through September 2020. We followed a systematic approach for contacting the patients using the last available registration address, the patients telephone numbers, the contact information (postal and telephone) of relatives or caregivers, and the patient primary care physician. All patients who were interviewed by telephone provided written informed consent to participate in the study. The study was approved by the University of Freiburg Ethics Committee (EK-Freiburg 553/19).

### Study endpoints and definitions

The primary endpoint of this study was long-term survival (Kaplan–Meier survival estimation) after hospital discharge. Secondary endpoints were 6- and 12-month survival rates as well as HRQL at the time of the follow-up (The Short-Form Health Survey-36 (SF-36) [14], St. Georges Respiratory Questionnaire (SGRQ) [15] and Hospital Anxiety and Depression Scale (HADS) [16]). In addition, predictors for hospital survival and 6-month survival of initial hospital survivors (landmark analysis) were investigated. The HRQL was compared with a sample of the German general population [17, 18]. The results of the respiratory questionnaire were compared with a chronic obstructive pulmonary disease (COPD) reference cohort [19] as well as with a sample of a European general population (IBERPOC, Spain) [20] (in absence of a representative German sample). In addition, we compared our results with the findings from previous ECMO (CESAR [7] and PRESERVE [8] study) and ARDS cohorts (meta-analysis of Dowdy et al*.* [21] and Herridge et al*.* [22]). Finally, we investigated the influence of the follow-up time and the duration of ECMO support on HRQL.

Successful ECMO weaning was defined as being free from ECMO support and alive for at least 48 h after decannulation. Unsuccessful weaning was defined as the inability to explant the ECMO device because of persistent respiratory failure or death during ECMO support or the need for re-cannulation within 48 h.

To evaluate the patients' disease severity, RESP [23], SOFA [24] and APACHE-II [25] scores as well as the p/F-ratio (paO2/FiO2) prior to cannulation were analysed.

Immunosuppression was defined as: immunosuppression in case of oncological malignancies (including haematooncological malignancies and active solid tumours), caused by the disease itself or by related therapy (chemotherapy or haematopoietic stem cell transplantation (HSCT) 12 months prior to ECMO support); immunosuppression in patients after solid organ transplantation; patients with autoimmune diseases and immunosuppressive therapies (cut-off for cortisone: ≥ 10 mg prednisolone equivalent) and patients with immunosuppression caused by HIV.

Furthermore, pulmonary pathogen spectrum ascertained by broncho-alveolar lavage and tracheal secretions was investigated. Assignment to pulmonary pathogens was based on concordance of microbiological findings with clinical signs of infection.

### ECMO centre and ECMO management

Our centre provides a 24/7 ECMO service and is localized joined to a 30-bed medical intensive care unit and part of a tertiary hospital. Typical numbers for veno-arterial and veno-venous cannulation are 65 and 35 per year, respectively.

In our institution, for mechanical ventilation (MV) in severe ARDS generally biphasic positive airway pressure (bilevel ventilation) is used. VV ECMO support was implemented in case of severe but potentially reversible respiratory failure, when lung-protective MV resulted in hypoxemia or hypercapnia following established criteria [26]. To date, lung-protective MV was defined as positive end expiratory pressure (PEEP) ≤ 15cmH_2_O, plateau pressure ≤ 30cmH_2_O, driving pressure ≤ 15cmH_2_O and FiO_2_ ≤ 50%. The management of vasopressors and fluid therapy was driven by clinical judgement of the ECMO experienced intensivist in charge and has been reported earlier [27]. Treatment algorithms and standard operating procedures were subject to optimizations during the observational period, reflecting current state-of-the-art recommendations and scientific knowledge. In particular, patient selection was adjusted with regard to comorbidities, so that patients with immunosuppression are only treated with ECMO after very careful evaluation and patients with lung fibrosis (with a few exceptions) are no longer supported with ECMO.

After initiation of VV ECMO, invasiveness of MV was reduced and ECMO flow was adjusted aiming for a peripheral oxygen saturation of 85–90% and partial pressure arterial oxygen of approximately 60 mmHg. Typical ventilator settings were: PEEP 15cmH_2_O, plateau pressure 25cmH_2_O, FiO_2_ 50%, respiratory rate 10/min. Details on ventilator management and prone positioning procedures have been described earlier [28]. Additional information about ECMO management is available in Additional file 1.

### Statistical analysis

Continuous variables are presented as median and interquartile range (IQR), categorical variables as numbers and percentages. Mann–Whitney U test was used for analysis of continuous variables, Pearson's Chi-squared test or Fisher’s exact test for categorical variables. Logistic regression analysis using forward selection with a threshold of p < 0.05 of all clinical characteristics (excluding survival prediction scores) was performed for predictors of hospital survival and 6-month survival. Results are given as odds ratio [(OR), 95% confidence interval (CI)], and a p value of ≤ 0.05 was considered statistically significant. Primary endpoint (long-term survival after hospital discharge) was analysed using the Kaplan–Meier method. Median follow-up time was calculated as the simple median time from discharge to last follow-up point. Statistical calculations were performed using IBM SPSS statistics 25.0 (Armonk, NY: IBM Corp, 2017). Survival analysis was conducted in R (R Core Team, 2014), and figures were produced using the package ggplot2 (Wickham, 2009) and GraphPad Prism 9 (San Diego, California USA, 2020).

## Results

### Patients and follow-up

A total of 289 patients were treated with VV ECMO at our centre in the study period (median age 55 (43–64) years, 67.1% males). These patients showed a high rate of underlying pulmonary diseases (30.1%), especially lung fibrosis (9%), and other comorbidities like immunosuppression (31%) and liver cirrhosis (7.6%, Table 1). Median SOFA score was 13 (10–15), APACHE-II score 26 (20.5–32) and RESP score 1 (–2–3) indicating a high disease severity.

**Table 1:** Clinical characteristics and their association to hospital survival

	All (*n* = 289)	Status after index hospitalisation			*p* value
		Alive (*n* = 129, 44.6%)		Dead (*n* = 160, 55.4%)	
Demographics					
Age (y)	55 (43–64)		53 (41.5–59.5)	56 (45–66.8)	**0.027**
Sex (male)	194 (67.1%)		89 (69%)	105 (65.6%)	0.545
BMI (kg/m^2^)	24.5 (23.4–29.3)		24.5 (22.9–30.2)	24.4 (23.5–27.8)	0.610
Underlying pulmonary disease	87 (30.1%)		32 (24.8%)	55 (34.4%)	0.078
COPD	25 (8.7%)		11 (8.5%)	14 (8.8%)	0.947
Asthma	16 (5.5%)		7 (5.4%)	9 (5.6%)	0.941
Lung fibrosis	26 (9%)		2 (1.6%)	24 (15%)	** < 0.001**
Cystic fibrosis	7 (2.4%)		1 (0.8%)	6 (3.8%)	0.102
LTOT	14 (4.8%)		3 (2.3%)	11 (6.9%)	0.073
Pulmonary hypertension	8 (2.8%)		1 (0.8%)	7 (4.4%)	0.064
Comorbidities					
Nicotine abuse	98 (33.9%)		50 (38.8%)	48 (30%)	0.118
Hypertension	99 (34.3%)		49 (38%)	50 (31.3%)	0.230
Diabetes mellitus	39 (13.5%)		17 (13.2%)	22 (13.8%)	0.888
CAD	36 (12.5%)		13 (10.1%)	23 (14.4%)	0.271
Chronic renal failure	21 (7.3%)		8 (6.2%)	13 (8.1%)	0.531
Chronic haemodialysis	2 (9.1%)		1 (12.5%)	1 (7.1%)	0.674
Liver cirrhosis	22 (7.6%)		4 (3.1%)	18 (11.3%)	**0.009**
Immunosuppression	89 (30.8%)		24 (18.6%)	65 (40.6%)	** < 0.001**
Oxygenation pre-ECMO					
FiO_2_ (%)	1 (0.8–1)		1 (0.8–1)	1 (0.8–1)	0.271
Horowitz index (mmHg)	72.5 (60.5–98.8)		77.1 (62.1–107)	70 (59.3–95.7)	0.256
D (A-a)O_2_ (mmHg)	556 (422.8–596.8)		550 (385.5–591.8)	570 (442.3–598)	0.115
Duration of MV before ECMO (d)	1.2 (0.3–3.5)		1.1 (0.2–3)	1.3 (0.3–5.3)	0.341
< 2 d	161 (59.6%)		76 (62.3%)	85 (57.4%)	0.418
2–7 d	69 (25.6%)		30 (24.6%)	39 (26.4%)	0.741
> 7 d	40 (14.8%)		16 (13.1%)	24 (16.2%)	0.475
Acute renal failure	95 (32.9%)		46 (35.7%)	49 (30.6%)	0.365
Scores					
SOFA score	13 (10–15)		12 (10–15)	13 (10–16)	0.439
APACHE-II score	26 (20.5–32)		25 (19–31)	27 (22–33)	**0.022**
RESP score	1 (-2–3)		2 (-0.5–4)	1 (-2–3)	**0.006**
Causes of ARDS					
Pneumonia	206 (71.3%)		89 (69%)	117 (73.1%)	0.440
Aspiration	25 (8.7%)		10 (7.8%)	15 (9.4%)	0.626
Other injuries	58 (20.1%)		30 (23.3%)	28 (17.5%)	0.225
Pulmonary pathogen spectrum					
Bacterial	120 (41.5%)		67 (51.9%)	53 (33.1%)	**0.001**
Viral	91 (31.5%)		44 (34.1%)	47 (29.4%)	0.389
Fungal	56 (19.4%)		16 (12.4%)	40 (25%)	**0.007**
Pneumocystis jirovecii	19 (6.6%)		4 (3.1%)	15 (9.4%)	**0.032**
Procedural characteristics and outcome					
ICU length of stay (d)	13.5 (9–23.5)		17.9 (11.7–32.8)	11.1 (5.5–18.9)	** < 0.001**
ECMO duration (d)	6.7 (3.9–12.1)		6.6 (4.4–11.5)	6.8 (3.3–13.2)	0.903
MV duration (d)	12.5 (7.6–22.4)		14.5 (9.5–30.2)	10.9 (5.3–19.4)	** < 0.001**
Dual-lumen cannula	245 (84.8%)		115 (89.1%)	130 (81.3%)	0.063
Primary non-IMV ECMO	18 (6.2%)		6 (4.7%)	12 (7.5%)	0.319
Tracheostomy	111 (38.4%)		62 (48.1%)	49 (30.6%)	**0.002**
Haemodialysis	109 (37.7%)		47 (36.4%)	62 (38.8%)	0.686

*p* values < 0.05 are presented in bold

APACHE II score: Acute Physiology And Chronic Health Evaluation; ARDS: acute respiratory distress syndrome; BMI: body mass index; CAD: coronary artery disease; COPD: chronic obstructive pulmonary disease; ECMO: extracorporeal membrane oxygenation; FiO_2_: fraction of inspired oxygen; ICU: intensive care unit; IMV: invasive mechanical ventilation; LTOT: long-term oxygen therapy; MV: mechanical ventilation; RESP score: Respiratory Extracorporeal Membrane Oxygenation Survival Prediction; SOFA score: Sequential Organ Failure Assessment

**Fig. 1:**
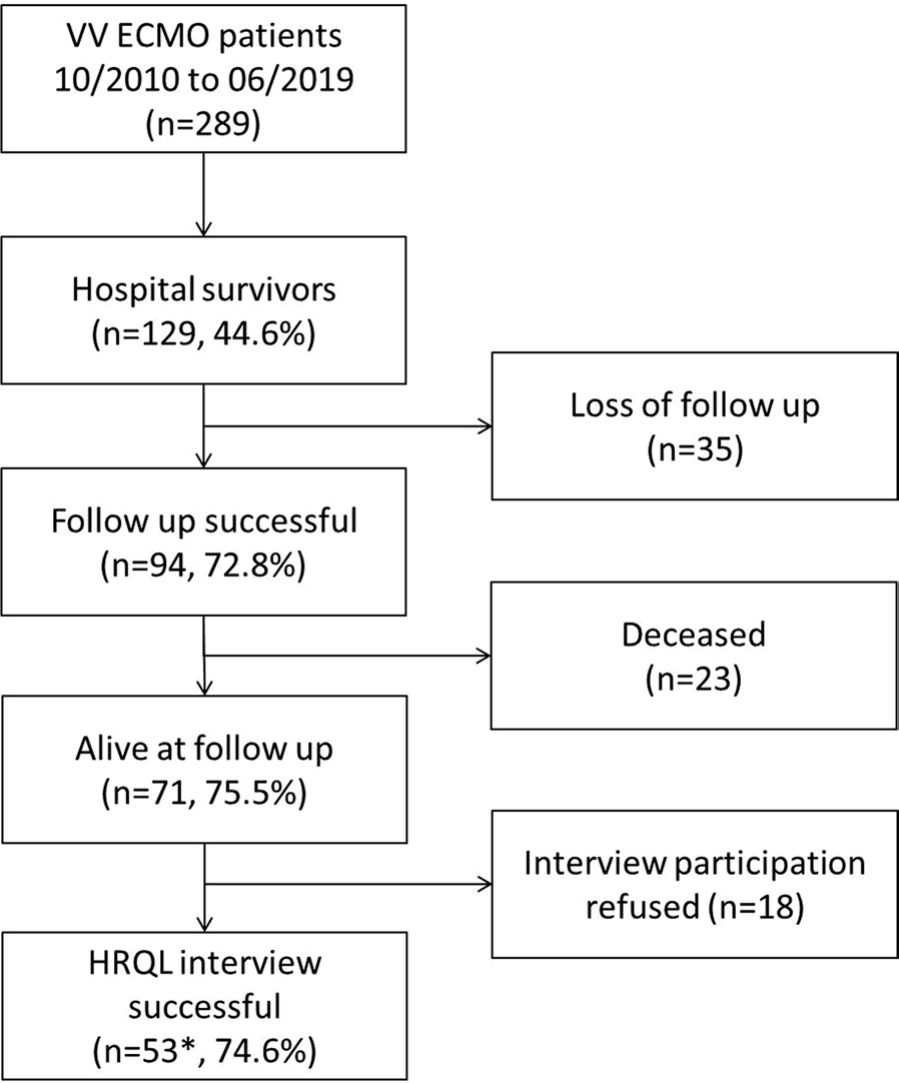
Study flow chart. *n = 53 completed SF-36 and *n* = 52 completed SGRQ and HADS

Follow-up duration ranged from 1.3 to 9.7 years with a median follow-up of 3.9 (2.2–6.6) years. Follow-up was successful in 94 of 129 hospital survivors (72.8%, Fig. 1). Seventy-one (75.5%) of these patients were alive at follow-up, and 53 patients (74.6%) agreed to a HRQL assessment.

### Hospital, mid-term and long-term survival

**Fig. 2:**
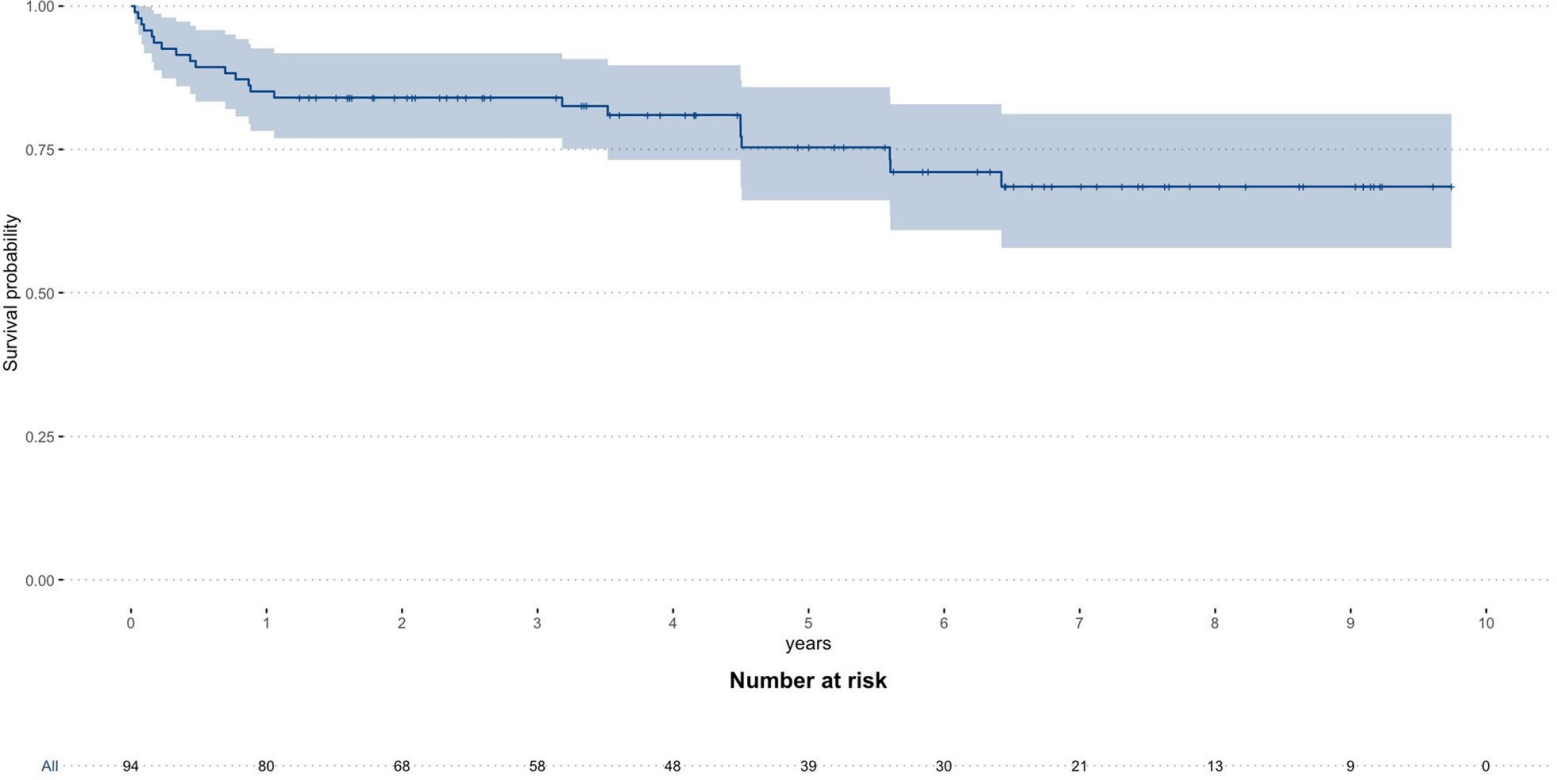
Long-term survival of VV ECMO hospital survivors. Kaplan–Meier survival estimation for all patients with VV ECMO in case of severe ARDS that survived the index hospital stay

Weaning was successful in 153 of 289 ECMO patients (52.9%) and 129 patients (44.6%) survived the index hospital stay. Hospital survivors showed a high mid-term survival rate with 84 of 94 patients (89.4%) alive after 6 months and 80 of 94 patients (85.1%) alive after 12 months, respectively. Kaplan–Meier estimation showed a survival rate of 68.5% (95%-CI 56.9–80.1%) 9.7 years after ECMO support (Fig. 2, Kaplan–Meier estimation of all patients is shown in Additional file 1: figure E6).

### Predictors for hospital and 6-month survival

**Fig. 3:**
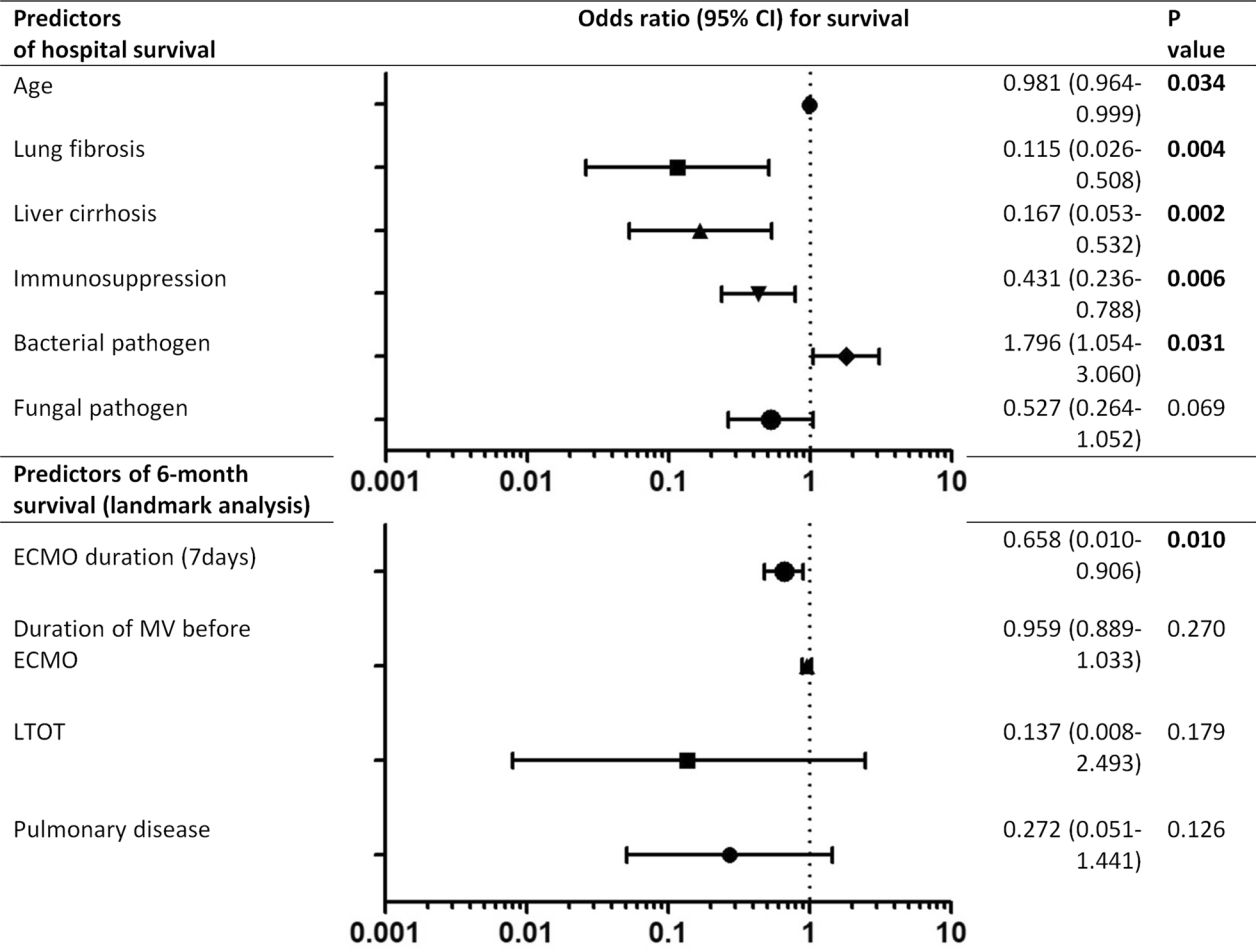
Predictors of hospital and 6-month survival. Logistic regression analysis of factor associated with hospital survival and with 6-month survival (6-month survival of primary hospital survivors—landmark analysis). Age, lung fibrosis, liver cirrhosis and immunosuppression were independent predictors for increased hospital mortality, while proof of bacterial infection was a predictor for increased survival. In the landmark analysis only the ECMO duration was an independent predictor for increased mortality. ECMO: extracorporeal membrane oxygenation; LTOT: long-term oxygen therapy; MV: mechanical ventilation.

In univariate analysis age, lung fibrosis, liver cirrhosis, immunosuppression, fungal pulmonary infection were associated with increased hospital mortality, while bacterial pulmonary infection was associated with increased hospital survival (Table 1). Logistic regression analysis revealed age, lung fibrosis, liver cirrhosis, immunosuppression and bacterial pulmonary infection as independent predictors for hospital mortality and survival, respectively (Fig. 3).

In the landmark analysis of hospital survivors with successful follow-up underlying pulmonary disease, long-term oxygen therapy, a duration of MV before ECMO of more than 7 days and the duration of ECMO support itself were associated with reduced 6-month survival (Additional file 1: Table E1). In logistic regression analysis only the duration of ECMO support was an independent predictor for 6-month mortality (odds ratio: 0.66 (95%-CI 0.01–0.91, p = 0.010) per week (Fig. 3).

### Long-term health-related quality of life

HRQL assessment was successful for 53 patients (one patient only completed SF-36, therefore 52 patients for SGRQ and HADS assessment) and conducted 3.9 (2.2–6.6) years after ECMO cannulation.

**Fig. 4:**
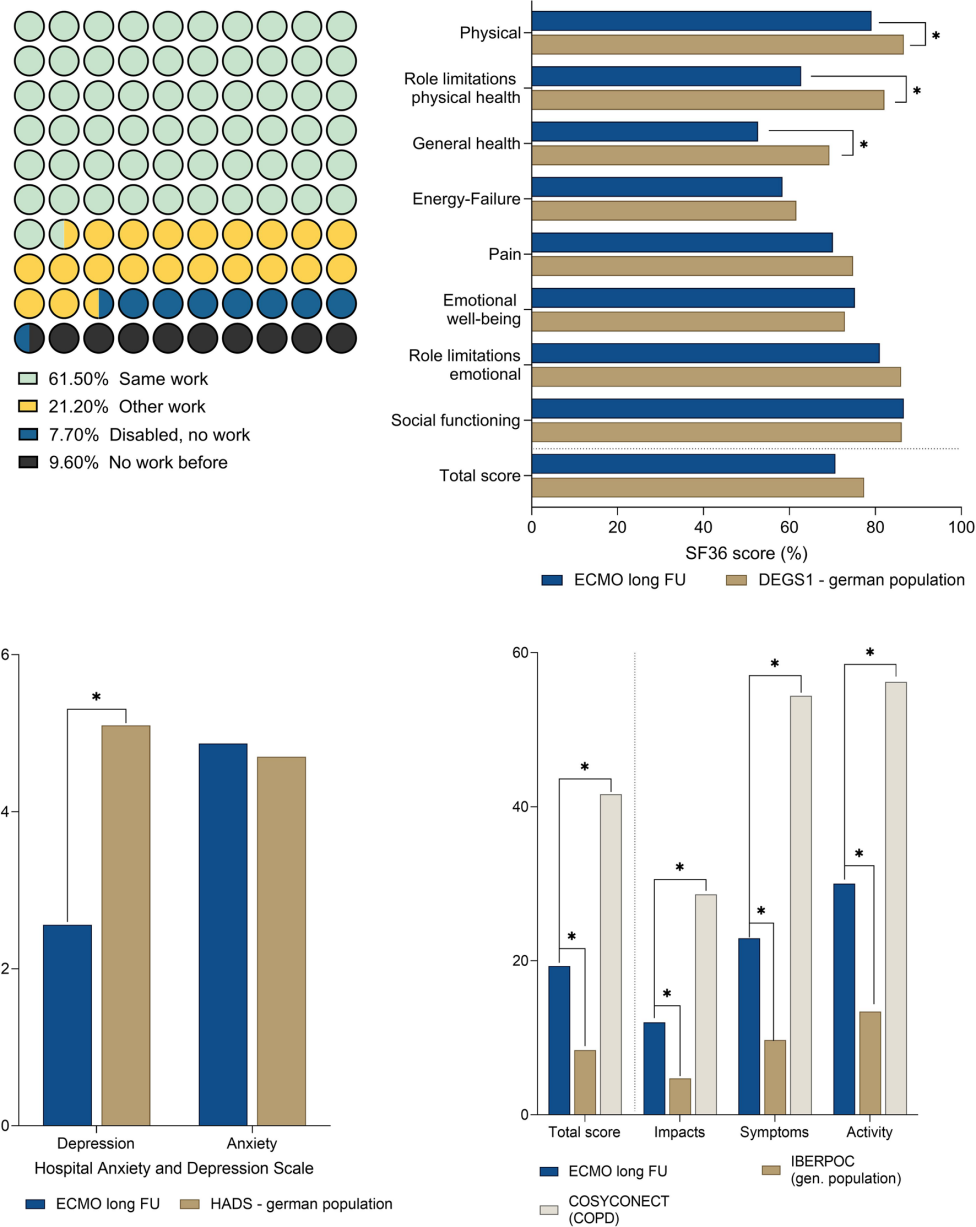
Health-related quality of life in the long-term follow-up of VV ECMO survivors. A) Distribution of patients, who were able to return to work after discharge, had to change their job or were no longer able to work. B) SF-36 of VV ECMO survivors compared to German general population (DESG1) [18]. Higher scores denote better health-related quality of life. C) HAD-D and HAD-A compared to German general population (Hinz et al*.*) [17]. Lower scores denote lower levels of depression and anxiety. D) SGRQ compared to the German COSYCONECT population (COPD reference cohort) [19] and the Spanish IBERPOC general population [20]. Lower scores denote lower levels of pulmonary impairment. *ECMO* extracorporeal membrane oxygenation; *VV* veno-venous

A great number of these patients were working at follow-up (82%; 61% continued in their previous job, 21% had to change their jobs), 8% were permanently disabled, and 10% were already without work before ECMO support (Fig. 4, a).

The SF-36 showed a high total score of 72.9 (61.7–83.8), which was within the range of the German age- and sex- adjusted reference cohort. Only the categories physical, role limitations, physical health and general health showed a higher level of limitations in the ECMO cohort (Fig. 4, b).

The level of anxiety (HAD-A) was comparable to the German reference cohort, and the level of depression (HAD-D) was even significantly lower in the ECMO cohort (Fig. 4, c).

Respiratory limitations (measured by the SGRQ) ranged between the limitations prevalent in the general population (IBERPOC) and those of a reference cohort of COPD patients (COSYCONECT). In every single category of the SGRQ (impacts, symptoms and activity), the patients of the ECMO cohort showed significant lower levels of limitation compared to the COPD cohort but higher levels of limitation compared to the general population (Fig. 4, d).

An association between HRQL and the time to follow-up after ECMO cannulation could not be demonstrated in this cohort (Additional file 1: Table E2). With the exception of the SF-36 physical role and HAD-A, there was also no association between HRQL and the duration of ECMO support (Additional file 1: Table E3).

Reference ECMO and ARDS cohorts showed comparable levels of HRQL. The SF-36 showed a slight trend in favour for the presented ECMO cohort in the total score and in the categories physical functioning, social functioning and emotional role (Additional file 1: figure E1 and E2). The results of the SGRQ and the HAD-A were comparable, while the results of the HAD-D were slightly higher in comparative groups (Additional file 1: figure E3 and E4). Moreover, there was a trend for a higher back to work rate in the presented ECMO patients compared to the reference studies (Additional file 1: figure E5).

## Discussion

This analysis describes, to the best of our knowledge, the longest follow-up period of VV ECMO patients reported so far and showed a remarkable long-term survival rate as well as high levels of health-related quality of life.

Patients in this cohort were similar in age and gender distribution to previous ECMO cohorts, but had a high rate of relevant comorbidities, particularly lung fibrosis, immunosuppression and liver cirrhosis, resulting in high hospital mortality.

Patients that survived initial hospitalisation showed a very high 6-month survival of nearly 90% which is comparable to the results of the CESAR trial [7]. Moreover, these patients showed a 10-year survival rate of approximately 70%.

Most analyses of survival predictors focus on a baseline analysis with respect to hospital survival or 6-month survival. To increase our understanding of factors that may affect post-discharge survival, we also performed a landmark analysis of hospital survivors. Predictors of hospital survival were age and severe pre-existing conditions such as lung fibrosis, liver cirrhosis, immunosuppression and pulmonary pathogen spectrum. These are typical factors which were associated with survival in previous ECMO studies [8, 23, 29–31] as well.

Most interestingly, our landmark analysis showed that pre-existing conditions of VV ECMO patients that survived the index hospitalisation were no longer associated with the probability of long-term survival. The only independent predictor of 6-month survival was the duration of ECMO support.

Possibly, patients with severe pre-existing conditions and a poor general state of health prior to ARDS die frequently during ECMO support and patients with less severe pre-existing conditions tend to survive. Therefore, these underlying diseases seem to play a minor role in the further course of the patients. In contrast, after the initial hospital survival the severity and course of the ARDS, represented by the duration of the necessary ECMO support, seems to play a more significant role in mid-term prognosis.

In addition to favourable long-term survival, the HRQL of these patients was also high.

In a comprehensive analysis of the quality of life by the SF-36, low extents of restrictions were shown in comparison with the age- and sex-adjusted German general population [18]. Interestingly, the only differences with lower quality of life were detected in general health and physical limitations. There were no limitations in the realm of the emotional situation and social functioning. In line with this, a specific analysis of the mental state of these patients did not show any restrictions compared to the German population [17]. In contrast, the level of the HAD-D scale, which indicates depression, was even below the level of the general population.

The evaluation of respiratory limitations, as measured by the SGRQ, was promising as well. Only moderate limitations were observed, which were intermediate between those of the general population [20] and a large population study of COPD patients [19].

Altogether, in a median of almost 4 years after ECMO, apart from minor physical limitations and moderate pulmonary limitation, the quality of life of these individuals was very high, especially with regard to their psychological condition.

The quality of life measured in our study cohort was even better than in previous HRQL analyses of ECMO or ARDS survivors [21, 32, 33]. One hypothesis could be a correlation between the time point of the HRQL survey and the level of remaining limitations. While the CESAR trial [7] with a 6-month follow-up reported a relatively low SF-36 score, the PRESERVE study [8] with an average follow-up a of 17 months reported better SF-36 scores. A similar distribution was found for the proportion of patients that were able to return to work. However, in this study there was no correlation between the duration of follow-up and the level of HRQL. This might be due to the fact that the shortest follow-up started at 1.3 years and thus the early phase after discharge could not be assessed. To further investigate this hypothesis, a serial prospective follow-up with standardized intervals would be necessary.

In summary, these results indicate a rather good HRQL after ECMO.

The median age of the patients in this study was 55 years, and they were therefore expected to continue to work for more than 10 years. Therefore, an economic consideration of the survivors is also important. Also from this point of view, the results were very encouraging, as only 8% of the patients who were working before the ARDS developed a disability and over 60% even were able to remain in their former job. This high rate of ability to work could be a result of the overall lower physical and mental limitations compared to previous studies [7, 8, 21].

In the here reported retrospective analysis, both survival and quality of life showed very encouraging long-term results. These results may help to strengthen the confidence of patients, relatives and ICU teams involved in the treatment of severe ARDS requiring ECMO support. In order to confirm these results and to explore changes in HRQL over time, large prospective studies with defined follow-up intervals should be conducted.

### Limitations

This is a retrospective observational study, and therefore, there is a risk of selection and reporting bias, although all ECMO patients of our centre were included and the ECMO indication was based on standardized algorithms. Thus, our patients showed similar disease severity and mortality compared to previous ECMO studies. Moreover, this is a single-centre report and centre-specific processes may influence the presented results. The loss of follow-up rate is comparable to previous studies on HRQL in ECMO patients, and we therefore consider it acceptable for a retrospective analysis and a particularly long follow-up period. However, a distortion of the results due to missing data (loss of follow-up 4 years after initial hospital stay was 27%) cannot be excluded. Moreover, one quarter of the patients did not participate in the HRQL interview. Together, due to these limitations, our findings should be considered as hypothesis-generating and should not prompt clinical decision-making.

## Conclusion

This analysis of VV ECMO patients showed an encouraging long-term survival rate with a high level of health-related quality of life and thereby demonstrates a promising perspective for ECMO survivors.

## Supplementary Information

**Additional file 1: Figure E1–E5 and Table E1–E3**: Long-term survival and health-related quality of life in patients with severe acute respiratory distress syndrome and veno-venous extracorporeal membrane oxygenation support—Online data supplement.

## Data Availability

The datasets used and/or analysed during the current study are available from the corresponding author on reasonable request.

## Abbreviations

APACHE II score: Acute Physiology And Chronic Health Evaluation
ARDS: Acute respiratory distress syndrome
BAL: Broncho-alveolar lavage
BMI: Body mass index
CAD: Coronary artery disease
COPD: Chronic obstructive pulmonary disease
D(A-a)O2: Alveolar-arterial gradient of oxygen concentration
ECMO: Extracorporeal membrane oxygenation
FiO_2_: Fraction of inspired oxygen
HRQL: Health-related quality of life
IMV: Invasive mechanical ventilation
ICU: Intensive care unit
LTOT: Long-term oxygen therapy
MV: Mechanical ventilation
PEEP: Positive end expiratory pressure
RESP score: Respiratory Extracorporeal Membrane Oxygenation Survival Prediction
SOFA score: Sequential Organ Failure Assessment
TS: Tracheal secretions
VV: Veno-venous
