# Supplementary material for: Long-term survival and health-related quality of life in patients with severe acute respiratory distress syndrome and veno-venous extracorporeal membrane oxygenation support
Source: Crit Care. 2021 Nov 29;25:410. doi: 10.1186/s13054-021-03821-0 (PMC8628468; doi:10.1186/s13054-021-03821-0)
Supplement: Supplementary file 1 — Additional file 1: Figure E1–E5 and Table E1–E3: Long-term survival and health-related quality of life in patients with severe acute respiratory distress syndrome and veno-venous extracorporeal membrane oxygenation support—Online data supplement. [file 13054_2021_3821_MOESM1_ESM.docx]

# Online data supplement

**Long-term survival and health-related quality of life in patients with severe acute respiratory distress syndrome and veno-venous extracorporeal membrane oxygenation support**

Jonathan Rilinger, Klara Krötzsch, Xavier Bemtgen, Markus Jäckel, Viviane Zotzmann, Corinna N Lang, Klaus Kaier, Daniel Duerschmied, Alexander Supady, Christoph Bode, Dawid L Staudacher, Tobias Wengenmayer

# Methods

**Additional information about ECMO centre and ECMO management**

Cannulations in our ECMO centre are performed by two experienced intensivists and a perfusionist in Seldinger’s technique without primary surgical cut down.

There is a 24/7 outreach team. Either SCPC (Sorin Centrifugal Pump Console, LivaNova, London, United Kingdom) or Cardiohelp (Maquet Getinge Group, Rastatt, Germany) ECMO systems were used.

Cannulation was performed predominately via jugular access using a dual-lumen cannula (Avalon (Maquet, Rastatt, Deutschland). For patients without life threatening bleeding, anticoagulation was provided by intravenous unfractionated heparin aiming at a partial thromboplastin time 1.5 times upper normal limit.

# Figures


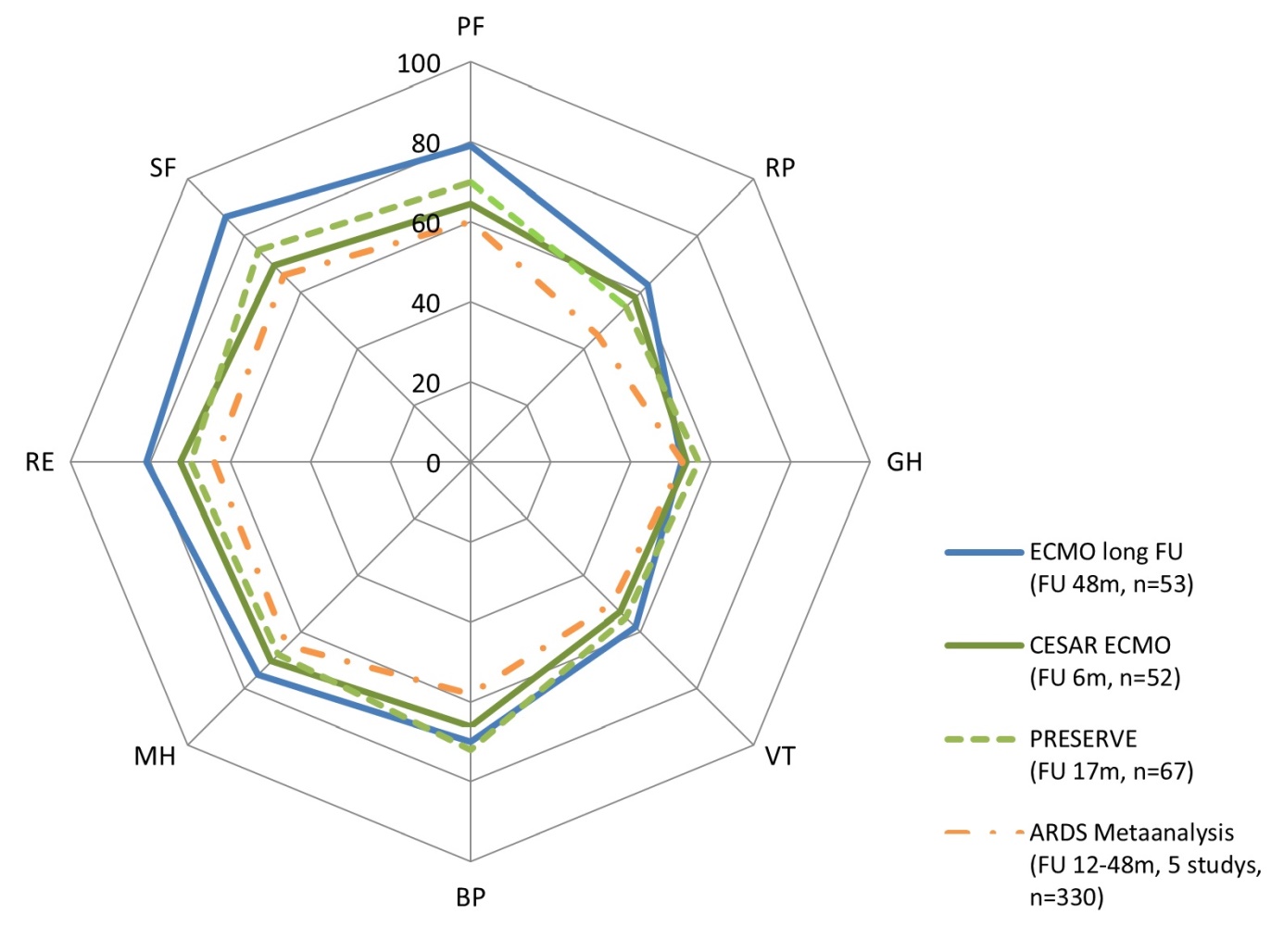


## Figure E1 Comparison of mean SF-36 with other ECMO cohorts

Mean SF-36 category scores were compared to the results of the CESAR [7] and PRESERVE [8] trial as well as to the study of Dowdy *et al.* [21]. Higher scores denote better health-related quality of life. *PF: Physical functioning; RP: Physical role; RE: Emotional role; VT: Vitality; MH: Mental health; SF: Social functioning; BP: Bodily pain; GH: General health;* *ECMO: extracorporeal membrane oxygenation; FU: follow up.*


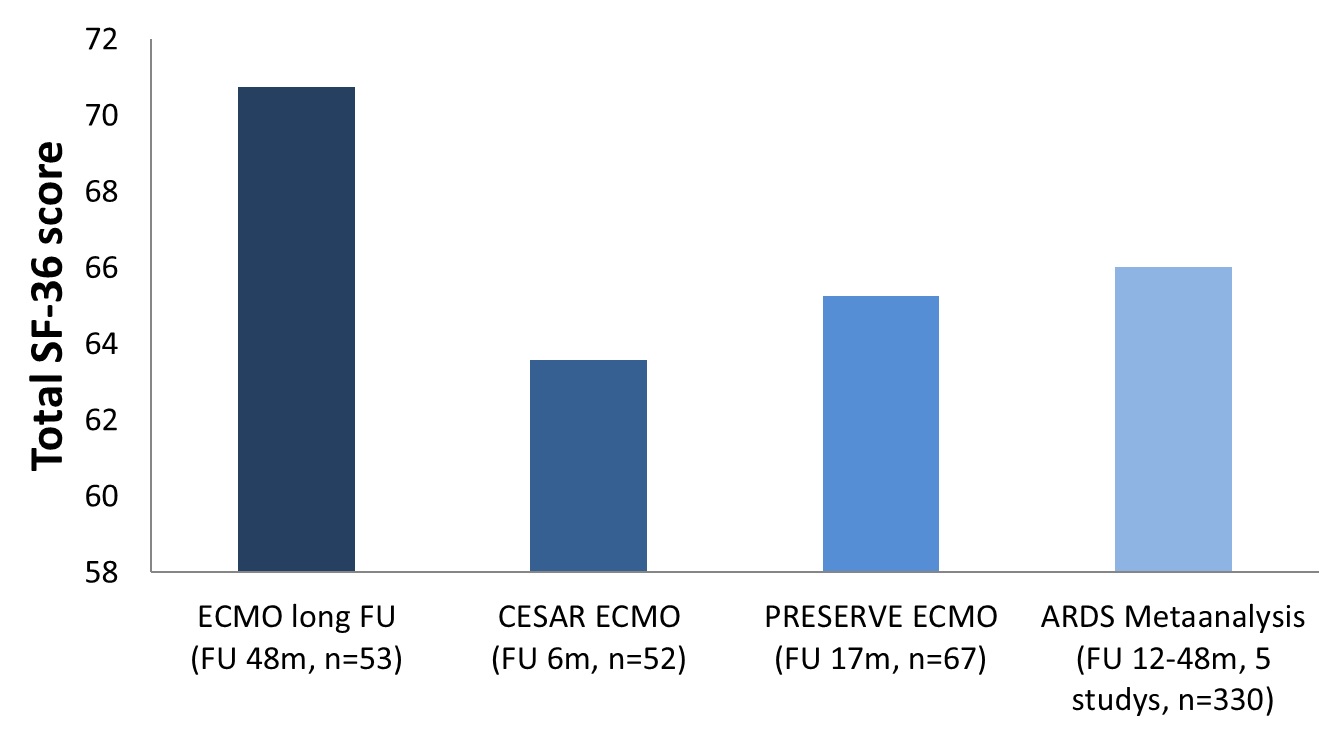


## Figure E2 Comparison of mean total SF-36 with other ECMO cohorts

Mean SF-36 total score was compared to the results of the CESAR [7] and PRESERVE [8] trial as well as to the ARDS meta-analysis of Dowdy *et al.* [21]. Higher scores denote better health-related quality of life. *ECMO: extracorporeal membrane oxygenation; FU: follow up.*


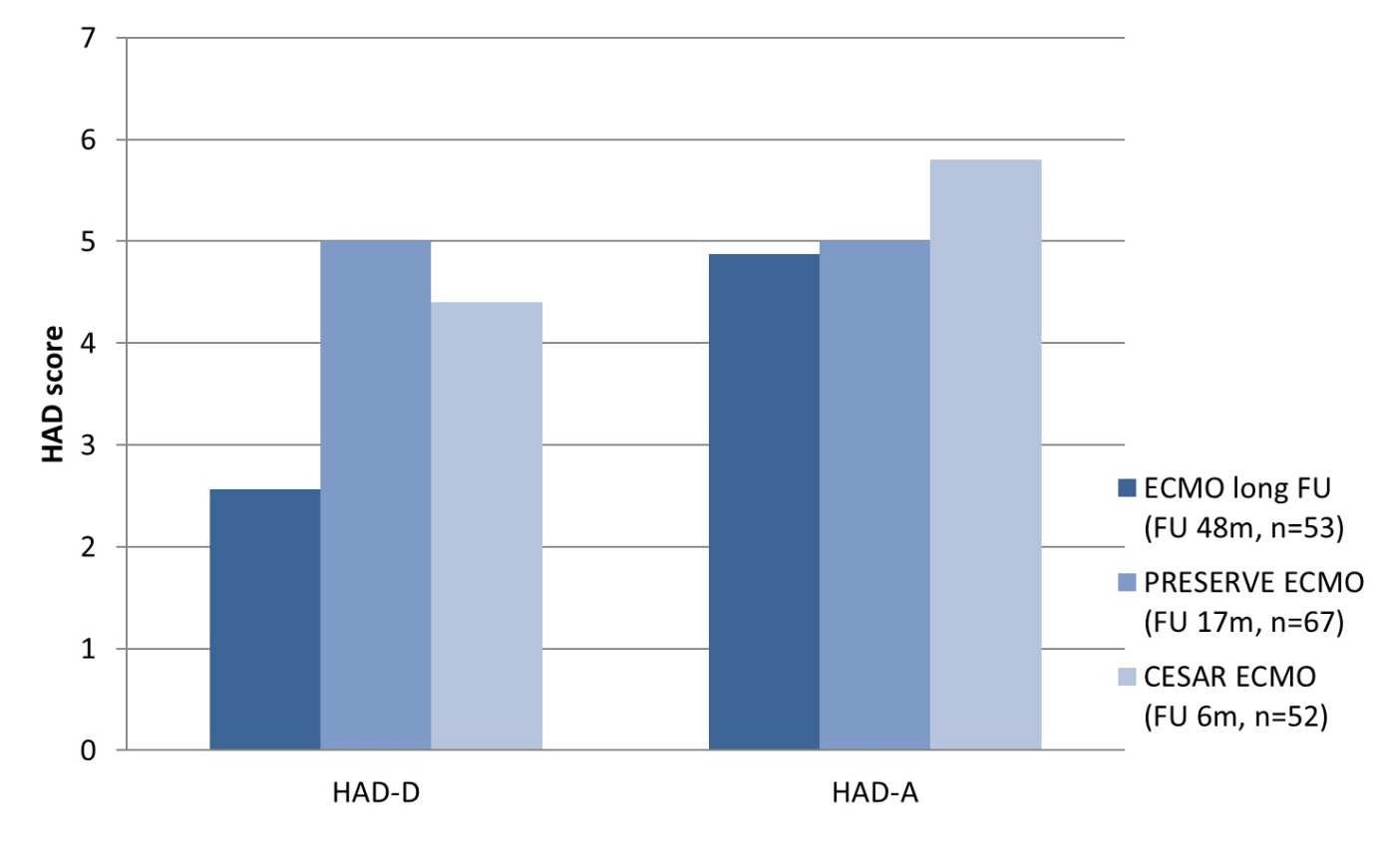


## Figure E3 Comparison of HAD-D and HAD-A score with reference cohorts

Mean HAD-D and HAD-A scores were compared to the results of the CESAR [7] and PRESERVE [8] trial. Lower scores denote lower levels of depression and anxiety. *ECMO: extracorporeal membrane oxygenation; FU: follow up.*


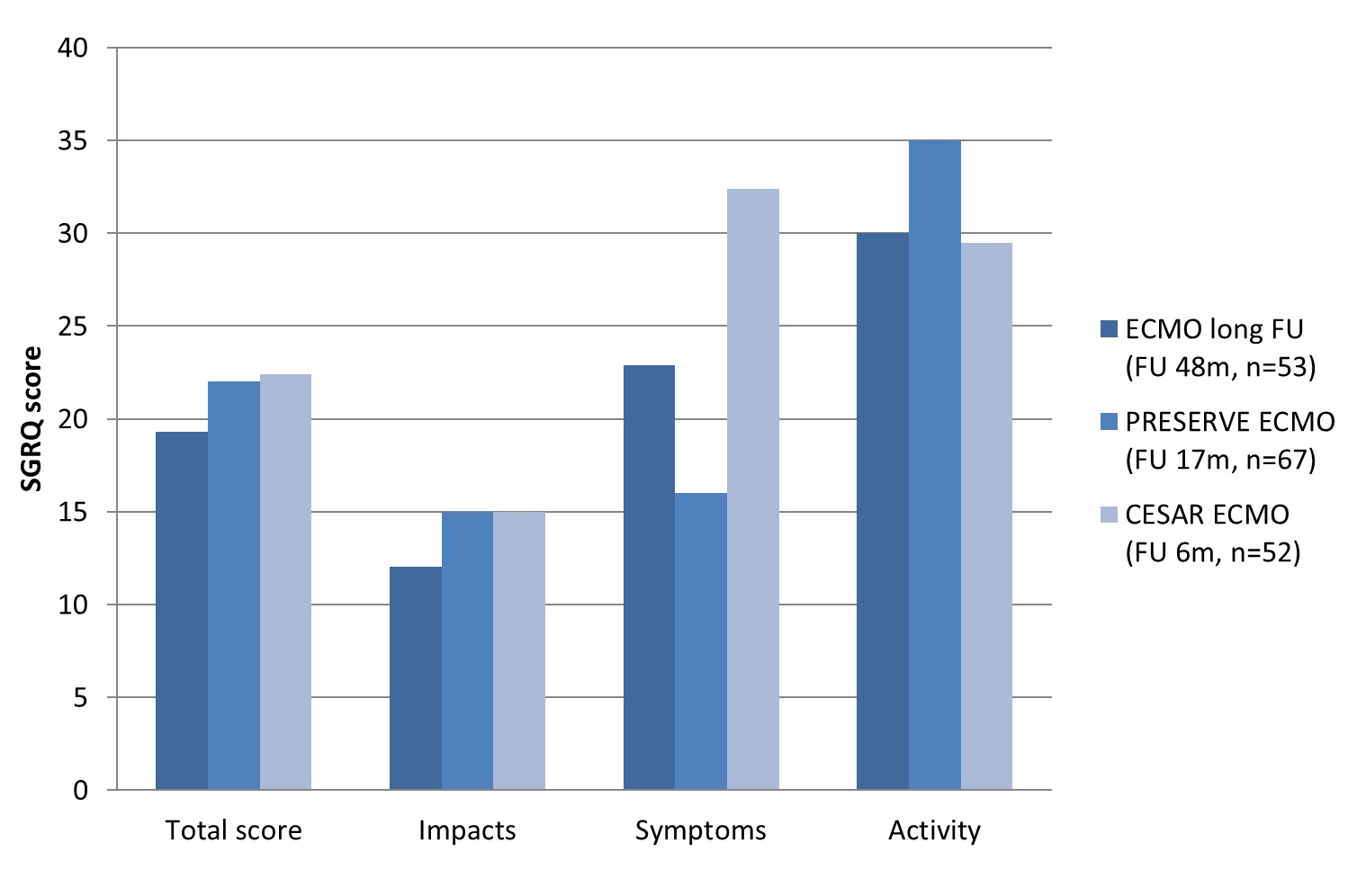


## Figure E4 Comparison of SGRQ with reference cohorts

Mean SGRQ scores and subscales were compared to the results of the CESAR [7] and PRESERVE [8] trial. Lower scores denote lower levels of pulmonary impairment. *ECMO: extracorporeal membrane oxygenation; FU: follow up.*


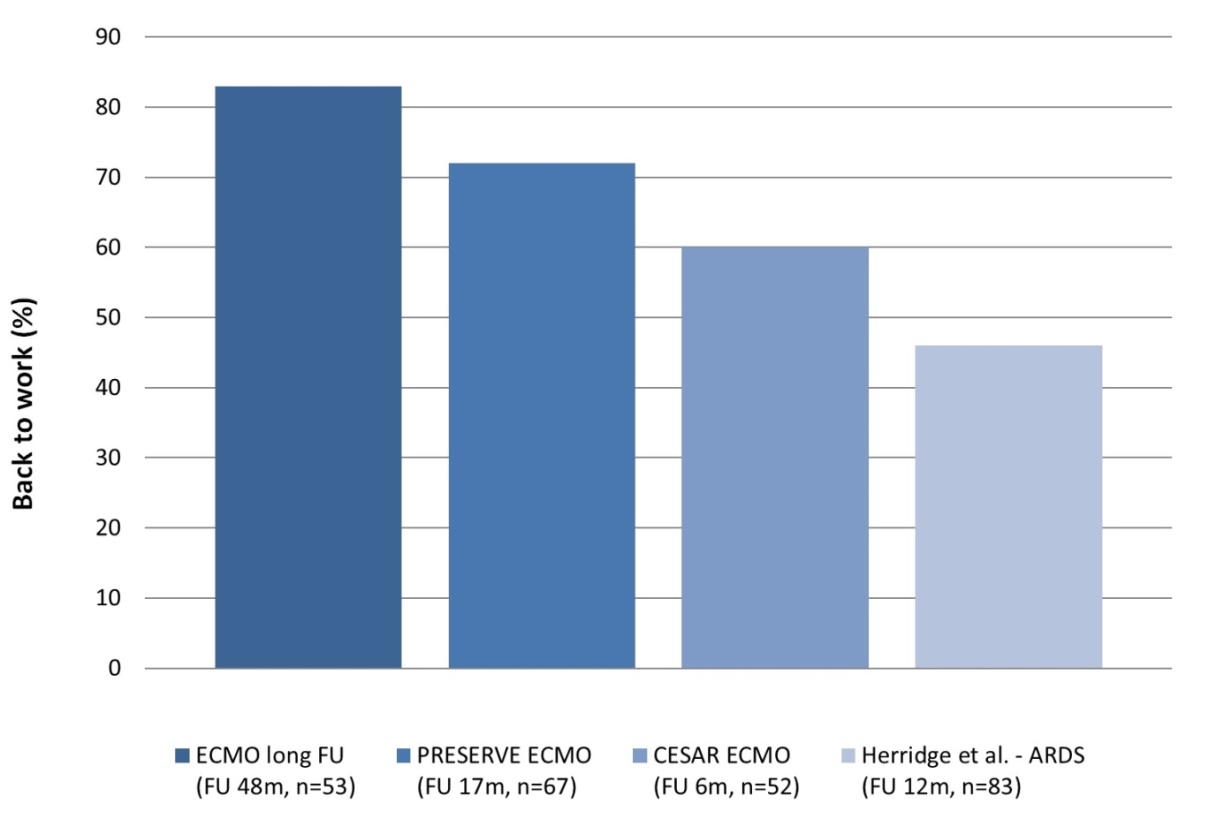


## Figure E5 Comparison of back to work rate with reference cohorts

Return to work rate was compared with the results of the CESAR [7] and PRESERVE [8] trial as well as to the study of Herridge *et al.* [22]. *ECMO: extracorporeal membrane oxygenation; FU: follow up.*


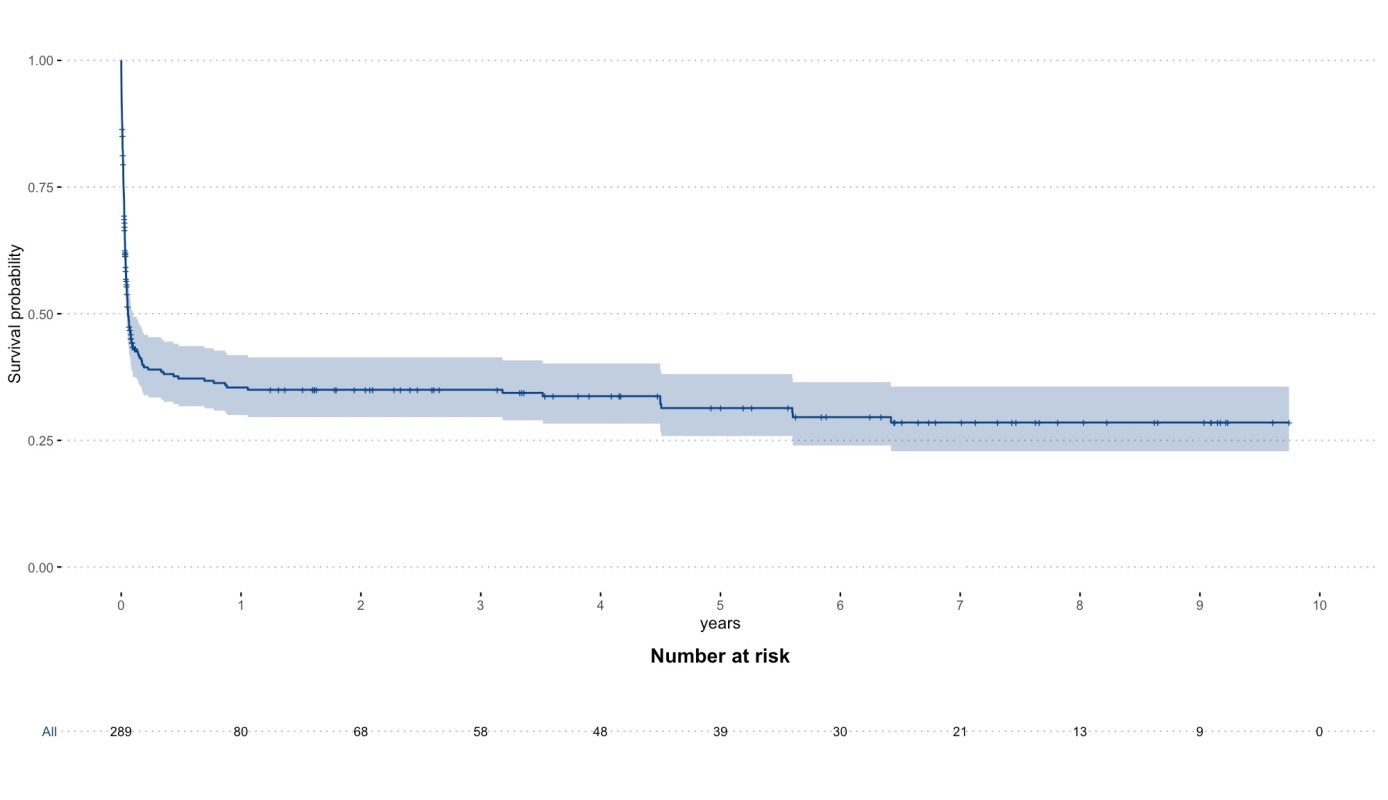


## Figure E6 Long-term survival of VV ECMO patients

Kaplan–Meier survival estimation for all patients with VV ECMO in case of severe ARDS (whole cohort).

# Tables

## Table E1. Univariate landmark analysis of hospital survivors*: Clinical characteristics at baseline and their association to 6 month survival

|  | **All**  **(n=94)** | **Status 6 months after ICU** | | | **P value** |
| --- | --- | --- | --- | --- | --- |
|  |  | **Alive**  **(n=84, 89.4%)** | | **Dead**  **(n=10, 10.6%)** |  |
| **Demographics** |  | |  |  |  |
| Age (y) | 55 (42-61) | | 54.5 (42-61) | 55.5 (53-73.5) | 0.320 |
| Sex (male) | 65 (69.1%) | | 56 (66.7%) | 9 (90%) | 0.131 |
| BMI (kg/m²) | 24.5 (22.9-31) | | 24.7 (23.2-32.2) | 23.7 (20.6-25.3) | 0.098 |
| Underlying pulmonary disease | 22 (23.4%) | | 17 (20.2%) | 5 (50%) | **0.036** |
| COPD | 5 (5.3%) | | 4 (4.8%) | 1 (10%) | 0.485 |
| Asthma | 5 (5.3%) | | 5 (6%) | 0 (0%) | 0.428 |
| Lung fibrosis | 1 (1.1%) | | 0 (0%) | 1 (10%) | 0.106 |
| Cystic fibrosis | 1 (1.1%) | | 0 (0%) | 1 (10%) | 0.106 |
| LTOT | 3 (3.2%) | | 1 (1.2%) | 2 (20%) | **0.029** |
| Pulmonary  hypertension | 0 (0%) | | - | - | - |
| Comorbidities |  | |  |  |  |
| Nicotine abuse | 33 (35.1%) | | 30 (35.7%) | 3 (30%) | 0.720 |
| Hypertension | 40 (42.6%) | | 36 (42.9%) | 4 (40%) | 0.863 |
| Diabetes mellitus | 14 (14.9%) | | 12 (14.3%) | 2 (20%) | 0.631 |
| CAD | 10 (10.6%) | | 8 (9.5%) | 2 (20%) | 0.310 |
| Chronic renal failure | 6 (6.4%) | | 6 (7.1%) | 0 (0%) | 0.382 |
| Chronic   haemodialysis | 0 (0%) | | - | - | - |
| Liver cirrhosis | 1 (1.1%) | | 1 (1.2%) | 0 (0%) | 0.729 |
| Immunosuppression | 17 (18.1%) | | 15 (17.9%) | 2 (20%) | 0.868 |
| Oxygenation pre ECMO |  | |  |  |  |
| FiO_2_ (%) | 1 (0.8-1) | | 1 (0.8-1) | 1 (0.9-1) | 0.341 |
| Horowitz index   (mmHg) | 76.9 (62.9-103.1) | | 77.4 (62.9-105.5) | 72.3 (61.8-86.9) | 0.559 |
| D(A-a)O_2_ (mmHg) | 548.5 (398.3-590.3) | | 548.5 (390.5-590.3) | 550 (447-593) | 0.761 |
| Duration of MV before  ECMO (d) | 1.1 (0.3-3.2) | | 1 (0.3-2.9) | 4.4 (0.9-13.5) | 0.057 |
| < 2 d | 54 (61.4%) | | 51 (63.7%) | 3 (37.5%) | 0.146 |
| 2-7 d | 22 (25%) | | 20 (25%) | 2 (25%) | 1.000 |
| > 7 d | 12 (13.6%) | | 9 (11.3%) | 3 (37.5%) | **0.039** |
| Acute renal failure | 31 (33%) | | 28 (33.3%) | 3 (30%) | 0.832 |
| In-house ECMO cannulation | 62 (66%) | | 55 (65.5%) | 7 (70%) | 0.775 |
| Scores |  | |  |  |  |
| SOFA score | 12 (10-15) | | 12 (10-15) | 12 (9.8-14) | 0.562 |
| APACHE-II score | 25 (17.8-31) | | 24.5 (17-30.8) | 30.5 (25-34.3) | **0.043** |
| RESP score | 2 (-0.3-4) | | 2 (0-4) | 0.5 (-3.5-3) | 0.156 |
| Causes of ARDS |  | |  |  |  |
| Pneumonia | 69 (73.4%) | | 60 (71.4%) | 9 (90%) | 0.209 |
| Aspiration | 6 (6.4%) | | 6 (7.1%) | 0 (0%) | 0.382 |
| Other injuries | 19 (20.2%) | | 18 (21.4%) | 1 (10%) | 0.395 |
| Pulmonary pathogen spectrum |  | |  |  |  |
| Bacterial | 49 (52.1%) | | 44 (52.4%) | 5 (50%) | 0.887 |
| Viral | 34 (36.2%) | | 32 (38.1%) | 2 (20%) | 0.260 |
| Fungal | 10 (10.6%) | | 10 (11.9%) | 0 (0%) | 0.248 |
| Pneumocysits  jirovecii | 3 (3.2%) | | 3 (3.6%) | 0 (0%) | 0.544 |
|  |  | |  |  |  |
| **Procedural characteristics** | | | | | |
| ICU length of stay (d) | 18.1 (11.2-33.2) | | 18 (11-32.8) | 27.4 (11.1-48.8) | 0.556 |
| ECMO duration (d) | 7.4 (4.7-12.9) | | 7.2 (4.7-11.8) | 14.4 (6.8-33) | **0.014** |
| MV duration (d) | 15.9 (9.6-32.3) | | 15.4 (9.1-31) | 27.9 (11.4-40.5) | 0.264 |
| Dual-lumen cannula | 83 (88.3%) | | 74 (88.1%) | 9 (90%) | 0.859 |
| Primary non IMV ECMO | 6 (6.4%) | | 4 (4.8%) | 2 (20%) | 0.062 |
| Tracheostomy | 46 (48.9%) | | 39 (46.4%) | 7 (70%) | 0.159 |
| Haemodialysis | 37 (39.4%) | | 33 (39.3%) | 4 (40%) | 0.965 |

*With successsful follow up.

*APACHE II score: Acute Physiology And Chronic Health Evaluation; ARDS: acute respiratory distress syndrome; BMI: body mass index; CAD: coronary artery disease; COPD: chronic obstructive pulmonary disease; ECMO: extracorporeal membrane oxygenation; FiO_2_: fraction of inspired oxygen; ICU: intensive care unit; IMV: invasive mechanical ventilation; LTOT: long-term oxygen therapy; MV: mechanical ventilation; RESP score: Respiratory Extracorporeal Membrane Oxygenation Survival Prediction; SOFA score: Sequential Organ Failure Assessment.*

## Table E2. HRQL divided by median follow up duration, 2 years of follow up and correlation between follow up duration and HRQL

| **Test** | **All patients**  **(n=53/52*)** | **<3.9 years FU**  **(n=26/25)** | **≥3.9 years FU**  **(n=25)** | **P value** | **<2 years FU**  **(n=10)** | **≥2 years FU**  **(n=43/42)** | **P value** | **Pearson correlation coefficient** | **P value** |
| --- | --- | --- | --- | --- | --- | --- | --- | --- | --- |
| **SF-36** |  |  |  |  |  |  |  |  |  |
| Physical functioning | 85 (67.5-95) | 85 (63.8-96.3) | 85 (70-95) | 0.900 | 67.5 (45-100) | 85 (75-95) | 0.238 | 0.086 | 0.540 |
| Physical role | 100 (12.5-100) | 100 (0-100) | 100 (25-100) | 0.746 | 12.5 (0-100) | 100 (25-100) | 0.119 | 0.106 | 0.450 |
| Emotional role | 100 (83.3-100) | 100 (66.7-100) | 100 (100-100) | 0.777 | 100 (50-100) | 100 (100-100) | 0.629 | 0.169 | 0.225 |
| Vitality | 60 (45-72.5) | 60 (48.8-80) | 55 (40-70) | 0.381 | 60 (45-80) | 60 (45-70) | 0.673 | -0.015 | 0.917 |
| Mental health | 76 (64-88) | 76 (64-88) | 80 (60-88) | 0.915 | 76 (58-84) | 76 (64-88) | 0.615 | 0.142 | 0.312 |
| Social functioning | 100 (75-100) | 100 (75-100) | 100 (100-100) | 0.470 | 100 (21.9-100) | 100 (75-100) | 0.454 | 0.195 | 0.162 |
| Bodily pain | 70 (45-100) | 80 (50-100) | 70 (45-100) | 0.474 | 50 (39.4-100) | 80 (45-100) | 0.208 | -0.002 | 0.989 |
| General health | 50 (40-67.5) | 50 (40-70) | 50 (35-60) | 0.436 | 60 (42.5-76.3) | 50 (40-60) | 0.213 | -0.131 | 0.348 |
| Total score | 72.9 (61.7-83.8) | 73.2 (62.1-82.7) | 72.9 (59.3-84.5) | 0.957 | 63.1 (53.6-81.7) | 73.2 (63.2-84.1) | 0.220 | 0.134 | 0.339 |
| **SGRQ** |  |  |  |  |  |  |  |  |  |
| Symptoms | 14  (6.6-38.1) | 13.2  (2.1-28.3) | 14.5  (8.8-45.7) | 0.155 | 14.8  (0-35.1) | 13.7  (6.7-39.5) | 0.429 | 0.166 | 0.239 |
| Activity | 35.6  (0-52.3) | 41.8  (0-56.9) | 17.1  (0-47.7) | 0.167 | 44.7  (0-71.3) | 26.8  (0-48) | 0.225 | -0.247 | 0.078 |
| Impacts | 7.1  (0.8-19.5) | 10.9  (0.4-16.8) | 5.5  (2-22.6) | 0.754 | 11.6  (0.4-18.8) | 6.3  (1.6-20.1) | 0.935 | -0.061 | 0.669 |
| total score | 19.4 (3.7-31.3) | 22 (2-28.7) | 8.6 (3.9-33.9) | 0.847 | 24.1 (1.8-34.4) | 16.4 (3.8-29.8) | 0.780 | -0.111 | 0.434 |
| **HADS** |  |  |  |  |  |  |  |  |  |
| HAD-D | 2 (1-3) | 2 (1-4) | 2 (1-3) | 0.881 | 1.5 (1-6.3) | 2 (1-3) | 0.669 | -0.062 | 0.664 |
| HAD-D ≥ 8 | 3 (5.8%) | 2 (8%) | 1 (3.7%) | 0.507 | 1 (10%) | 2 (4.8%) | 0.523 |  |  |
| HAD-A | 3 (2-7.8) | 3 (2-8) | 5 (2-8) | 0.506 | 3 (1-10) | 3 (2-7.3) | 0.752 | 0.091 | 0.520 |
| HAD-A ≥ 8 | 13 (25%) | 6 (24%) | 7 (25.9%) | 0.873 | 3 (30%) | 10 (23.8%) | 0.685 |  |  |

*1 patient had not completed SGRQ and HADS. *FU: follow up.*

## Table E3. HRQL divided by median ECMO duration, 14 day cut of ECMO duration and correlation between ECMO duration and HRQL

| **Test** | **All patients**  **(n=53/52*)** | **ECMO < 6.7d**  **(n=26)** | **ECMO ≥ 6.7d**  **(n=27/26)** | **P value** | **ECMO < 14d**  **(n=44/43)** | **ECMO ≥ 14d**  **(n=9)** | **P value** | **Pearson correlation coefficient** | **P value** |
| --- | --- | --- | --- | --- | --- | --- | --- | --- | --- |
| **SF-36** |  |  |  |  |  |  |  |  |  |
| Physical functioning | 85 (67.5-95) | 85 (72.5-95) | 80 (65-95) | 0.879 | 85 (66.3-95) | 75 (65-87.5) | 0.161 | -0.154 | 0.271 |
| Physical role | 100 (12.5-100) | 100 (25-100) | 75 (0-100) | 0.340 | 100 (25-100) | 0 (0-75) | **0.039** | -0.304 | **0.027** |
| Emotional role | 100 (83.3-100) | 100 (66.7-100) | 100 (100-100) | 0.777 | 100 (75-100) | 100 (66.7-100) | 0.935 | -0.167 | 0.233 |
| Vitality | 60 (45-72.5) | 60 (43.8-71.3) | 60 (45-75) | 0.661 | 60 (41.3-73.8) | 60 (55-72.5) | 0.407 | -0.034 | 0.809 |
| Mental health | 76 (64-88) | 74 (63-84) | 80 (64-88) | 0.406 | 76 (64-87) | 84 (64-92) | 0.536 | -0.058 | 0.681 |
| Social functioning | 100 (75-100) | 100 (75-100) | 100 (100-100) | 0.513 | 100 (75-100) | 100 (87.5-100) | 0.753 | -0.033 | 0.814 |
| Bodily pain | 70 (45-100) | 75 (49.4-100) | 57.5 (45-100) | 0.629 | 85 (45.6-100) | 57.5 (45-85) | 0.368 | -0.144 | 0.305 |
| General health | 50 (40-67.5) | 47.5 (35-60) | 55 (40-70) | 0.128 | 50 (36.3-68.8) | 50 (42.5-67.5) | 0.825 | 0.211 | 0.129 |
| Total score | 72.9 (61.7-83.8) | 73.4 (58.9-82.3) | 72.6 (62.4-85.5) | 0.695 | 73.9 (60-84.4) | 65.1 (61.9-77.2) | 0.246 | -0.201 | 0.148 |
| **SGRQ** |  |  |  |  |  |  |  |  |  |
| Symptoms | 14 (6.6-38.1) | 9.5 (4.4-30.5) | 18.9 (6.7-47.7) | 0.169 | 13.7 (4.4-38.7) | 18.8 (7.8-52.1) | 0.584 | 0.262 | 0.061 |
| Activity | 35.6 (0-52.3) | 15.2 (0-53.6) | 41.7 (0-50.2) | 0.388 | 24 (0-47.7) | 47.7 (8.6-57.6) | 0.240 | 0.212 | 0.131 |
| Impacts | 7.1 (0.8-19.5) | 4.6 (1.6-14.2) | 13.3 (0.4-21.5) | 0.507 | 7.1 (2-19.7) | 10.9 (0.4-19.6) | 0.905 | 0.093 | 0.514 |
| total score | 19.4 (3.7-31.3) | 8.4 (3.3-31.1) | 26.2 (4-32) | 0.370 | 14.8 (3.6-31.4) | 22 (6.3-34.9) | 0.651 | 0.2 | 0.156 |
| **HADS** |  |  |  |  |  |  |  |  |  |
| HAD-D | 2 (1-3) | 2 (1-3.3) | 1.5 (1-3.3) | 0.549 | 2 (1-4) | 2 (1-3) | 0.757 | -0.009 | 0.947 |
| HAD-D ≥ 8 | 3 (5.8%) | 2 (7.7%) | 1 (3.8%) | 0.552 | 3 (7.0%) | 0 (0%) | 0.414 |  |  |
| HAD-A | 3 (2-7.8) | 3 (2-8) | 3 (2-7.5) | 0.605 | 5 (2-8) | 2 (1.5-3) | **0.023** | -0.123 | 0.385 |
| HAD-A ≥ 8 | 13 (25%) | 7 (26.9%) | 6 (23.1%) | 0.749 | 13 (30.2%) | 0 (0%) | 0.057 |  |  |

*1 patient had not completed SGRQ and HADS. *FU: follow up.*
